# Natural microbial communities supporting the transfer of the IncP-1β plasmid pB10 exhibit a higher initial content of plasmids from the same incompatibility group

**DOI:** 10.3389/fmicb.2014.00637

**Published:** 2014-11-24

**Authors:** Xavier Bellanger, Hélène Guilloteau, Bérengère Breuil, Christophe Merlin

**Affiliations:** Laboratoire de Chimie Physique et Microbiologie pour l’Environnement, UMR 7564 Université de Lorraine – Centre National de la Recherche Scientifique, NancyFrance

**Keywords:** plasmid transfer, IncP-1, pB10, superinfection immunity, community permissiveness

## Abstract

Antibiotic resistance gene transfer mediated by plasmids is a matter of concern for public health, but permissive environments supporting plasmid dissemination are still quite difficult to identify. Lately, we have reported a molecular approach based on quantitative PCR (qPCR) to monitor the fate of the IncP-1β plasmid pB10 in natural microbial communities maintained in microcosms. Such plasmid transfer experiments were carried out with 13 different environmental matrices, and demonstrated that the transfer of the conjugative-proficient plasmid pB10 in complex environments is relatively rare and is strongly matrix dependent. An attempt to link the microbial community structure and the matrix permissiveness showed that TTGE analysis is not resolutive enough to point out common features among comparable communities supporting pB10 transfer. However, an estimation of the IncP-1α/IncP-1β plasmids abundance by qPCR demonstrated that pB10 transfer tends to be supported by environmental matrices exhibiting a higher content of IncP-1 plasmids. We suggest that the relative abundance of IncP-1 plasmids in a given microbial community reflects its permissiveness to the transfer of plasmids belonging to the same incompatibility group, which prevails over transfer limitation due to a phenomenon known as superinfection immunity.

## INTRODUCTION

Bacteria have evolved various adaptive mechanisms allowing them to deal with the constraints imposed by their ever-changing local environments. The astonishing genetic plasticity observed in prokaryotes clearly shows how these microorganisms innovate and adapt when challenged by novel environmental stresses such as the exposure to xenobiotics for instance (Heuer et al., 2008; Toussaint and Chandler, 2012). For part, the bacterial genetic flexibility is associated with mobile genetic elements that seem to play a significant role in acquiring and sharing new genes, enabling bacteria to improve their fitness under local stresses. In this respect, the dissemination of resistance genes that followed (and still follows) the use and consumption of antibiotics is just a dramatic illustration of such adaptability (Davies, 1995, 2007; Davies and Davies, 2010; Wiedenbeck and Cohan, 2011). Because mobile genetic elements obviously provide a selective advantage with the accessory genes they mobilize, their relative abundance is believed to increase together with the level of stress surrounding the cognate bacterial community (Kristiansson et al., 2011; Stalder et al., 2012).

The threat posed by the dissemination of antibiotic resistance genes has motivated a number of studies aiming to identify environmental hotspots displaying high occurrences of resistance genes. These studies clearly showed that, in addition to medical settings, some environments such as wastewater treatment plants or farms for instance, also displayed elevated levels of antibiotic resistance genes (Wright, 2010; Rizzo et al., 2013), and that receptor environments such as soil could even accumulate resistance genes over time (Knapp et al., 2010). The risk of having these environments spreading antibiotic resistances by horizontal transfer is further fueled by the circumstantial evidences showing that an increasing abundance of resistance genes could be concomitantly associated with an increasing abundance of mobile genetic elements including integrons and plasmids (Heuer et al., 2009; Kristiansson et al., 2011; Wang et al., 2013; Port et al., 2014). Although expected, there is no direct demonstration showing that a higher abundance of mobile genetic elements means that more horizontal transfer could take place. Indeed, it could be argued that resistant bacteria hosting mobile genetic elements could accumulate in some environments that are not necessarily permissive to horizontal gene transfer.

In this work, we focused on parameters that make a given bacterial community permissive to plasmid transfer (or not). A set of 13 different environmental communities, used as recipient, were assessed for their ability to support the transfer of the broad host range IncP-1β plasmid pB10 (Schlüter et al., 2003). To do so, we used a molecular method based on quantitative PCR (qPCR) that allows detecting relatively rare successful transfer events while working with low inocula of donor bacteria (Bonot and Merlin, 2010; Merlin et al., 2011; Bellanger et al., 2014). Basically, it consists in monitoring the relative abundance of both the plasmid and the donor DNAs over time after inoculation in total community DNA. Because conjugative transfer is an intercellular mode of replication, the plasmid to donor ratio should increase if successful transfer events occurred. Using this new approach, we could demonstrated the transfer of plasmid pB10 in microbial communities collected from different environmental matrices including river sediments or spatially organized communities of fixed biofilm reactors in wastewater treatment plants (Bonot and Merlin, 2010; Merlin et al., 2011). Additionally, we could show that environmental parameters, such as low level of dissolved oxygen or sublethal concentrations of antibiotics, could increase the persistence of pB10 in communities, while other parameters could affect pB10 dissemination as seen with the eukaryotic predation (Merlin et al., 2011; Bellanger et al., 2014). Taking advantage of having related communities behaving opposingly with respect to pB10 transfer, we attempted to point out specific community characteristics using classical molecular ecology methods. Then, the lack of recognizable community structure able to explain the absence (or presence) of pB10 transfer led us to investigate the relationship between the permissiveness to pB10 transfer and the IncP-1 plasmid content of the communities. Indeed, because “superinfection immunity” has been extensively described *in vitro* as a limiting factor for plasmid transfer (Garcillán-Barcia and de la Cruz, 2008), the various matrices supporting or not the transfer of pB10 were re-analyzed for their content in plasmids of the same incompatibility group (IncP-1).

## MATERIALS AND METHODS

### ENVIRONMENTAL MATRICES AND MICROCOSM SET UP

Some of the environmental matrices used in this study were described previously: sediments from the Moselle river (Bonot and Merlin, 2010); cow (Prim’Holstein) manure (Bellanger et al., 2014); activated sludge, recirculation sludge and effluents collected from the cattle slaughterhouse WWTP [#3] (Bellanger et al., 2014); and biofilms from a moving bed reactor collected in a municipal WWTP [#5] (Merlin et al., 2011). Only activated sludge, recirculation sludge and effluents collected from two additional cattle slaughterhouse WWTPs [#1] and [#2] and the municipal WWTP [#4] were new to this study, and were treated as the WWTP samples mentioned above. Additional information regarding the sampling sites can be found in the supplementary material.

The different environmental matrices sampled were used in microcosm experiments to assess their propensity to support the transfer of plasmid pB10. The microcosm setups and operations have already been described by Bonot and Merlin (2010) for river sediments, by Merlin et al. (2011) for municipal WWTP samples, and by Bellanger et al. (2014) for slaughterhouse WWTP samples and manure. In all cases, the environmental matrices were used fresh. Transfer experiments started by inoculating the donor bacteria *Escherichia coli* DH5α(pB10) (Schlüter et al., 2003) at a final concentration of 2.10^5^ cfu/mL (or 10^7^ cfu/mL for manure microcosms). The *E. coli* DH5α(pB10) inoculum was prepared by overnight culture in LB medium supplemented with 10 μg/mL of tetracycline (at 30°C under agitation at 160 rpm), followed by two cell washes alternating centrifugation and cell dispersion in 10 mM MgSO_4_ before inoculation. Microcosms were sampled at intervals and concentrated by centrifugation. Pellets (typically 1–1.5 g) were dispersed in one volume of the remaining supernatant (ca. 5 mL), and frozen at -80°C prior to community DNA extraction. The abundance of both plasmid pB10 and *E. coli* DH5α DNA was monitored by qPCR on community DNAs. For each plasmid transfer experiment two types of control were carried out in parallel. First, sets of non-inoculated control microcosms were systematically run in parallel in order to rule out any natural occurrence or resurgence of non-specific pB10-like DNA sequences in the subsequent qPCR analyses. Second, considering the fact that DNA released from the donor strain might remain detectable by qPCR, an additional set of controls was run for all the environmental matrices by inoculating replicate microcosms with naked DNA instead of DH5α(pB10) cells. In all cases, the levels of DNA detected by qPCR after inoculation with naked DNA were far below those obtained for the cognate microcosms inoculated with *E. coli* DH5α(pB10) (by at least 2 logs for equivalently sized inocula), therefore ruling out (i) the incidence of any possible DNA release from the donor bacteria in the phenomena observed, and (ii) the significance of any natural transformation by pB10 in the transfer observed.

### TOTAL DNA EXTRACTION FROM ENVIRONMENTAL SAMPLES

Total community DNAs were extracted from 500 mg (wet weight) of thawed environmental sample (manure, sediment, or sludge) following the procedure described by Porteous et al. (1997) later modified by Bonot et al. (2010). For liquid WWTP effluents, community DNAs were extracted from cell pellets collected by centrifugation of 25 mL samples, using the “Wizard Genomic DNA Purification Kit” (Promega) according to the recommendations delivered by the manufacturer. DNA concentration and purity were estimated spectrophotometrically based on absorbance readings at 230, 260, and 280 nm, using a BioPhotometer^TM^ (Eppendorf): A_260_ was used for calculation of DNA concentration, while ratios A_260/280_ and A_260/230_ were used for estimating DNA purity. The absence of residual inhibitors in the DNA extracts was checked by qPCR after spiking known quantities of target DNA and/or by serially diluting the template prior amplification.

### QUANTITATIVE PCR ASSAYS

The abundance of plasmid pB10 and *E. coli* DH5α DNA were estimated by qPCR using highly specific Taqman^®^ probes (Applied Biosystems) and primers (Eurogentec SA; **Table 1**). As previously explained (Bonot and Merlin, 2010), the specificity the qPCR carried out lies on the genetic organization of the targets rather that the uniqueness of their sequence. Basically, sets of qPCR primers were designed to prime on both sides of a unique junction between building blocks of the element targeted for quantification: a unique assembly between two truncated transposons for plasmid pB10 (target coordinates 45968–46102; GenBank NC_004840), and both sides of a unique 97 kb-deletion in the chromosome of *E. coli* DH5α (target coordinates on K12 274654–372933; GenBank U00096; Bonot and Merlin, 2010; Merlin et al., 2011). qPCRs were performed in triplicate using a “Step One Plus Real-Time PCR System” (Applied Biosystems, driver: StepOne Software v2.2) with thermocycling conditions set as follows: 2 min at 50°C, then 10 min at 95°C followed by 45 cycles of 15 s at 95°C and 1 min at 60°C. Quantifications were carried out from 25 ng of community DNA using a “TaqMan^®^ Universal PCR Master Mix, NoAmpErase^®^ UNG” (Applied Biosystems) as recommended by the manufacturer, with 800 nM of each primer, and 300 nM of TaqMan probe, in a 25 μL reaction volume. All data were normalized and expressed as a number of copies per μg of community DNA. Except for manure microcosm experiments, standard curves correlating the detection threshold of the fluorescence signal (Cq) to known concentrations of target DNA were generated by qPCR on pure template DNA obtained as follows: pB10 was extracted from *E. coli* DH5α(pB10) using a Wizard^®^Plus SV Minipreps kit (Promega, Madison, WI, USA), DNA was then linarized by digestion with *Bam*HI (Promega), and repurified using a QIAquick^®^PCR purification kit (Qiagen). Genomic DNA from DH5α was extracted using the AquaPure Genomic DNA isolation kit (Bio-Rad). For manure microcosm experiments, plasmid pBELX digested by *Bam*HI was used as standard. Plasmid pBELX is a standard pEX-A vector containing a synthetic sequence (Eurofins, France) covering the pB10 and DH5α DNA target sequences (**Table 1**), and for which no difference could be observed in terms of qPCR efficiencies compared to the standard curves made with the native pB10 and DH5α DNAs. Most of the time, the concentrations of either pB10 or *E. coli* DH5α DNAs in microcosms evolved over time following a log-normal function, with a disappearance rate (expressed in “Log per day”) that could be calculated from an exponential curve fitting. A slower disappearance rate of pB10 compared to *E. coli* DH5α DNA results in an increasing plasmid to donor ratio, signifying that plasmid transfer took place.

**Table 1:** Primers and probes used for PCR and qPCR.

Name	Sequences	Purpose	Reference
P1	5′-CAATACCGAAGAAAGCATGCG-3′	Quantification of pB10 by	Bonot and Merlin (2010)
P2	5′-AGATATGGGTATAGAACAGCCGTCC-3′	qPCR (amplicon: 135 bp)	
S1^a^	(FAM)5′-CCTCCACGGTGCGCGCTG-3′(TAMRA)		

P3	5′-ACCGGGTACATCATTTCC-3′	Quantification of DH5α by	Bonot and Merlin (2010)
P4	5′-GCCCCGGTAAGAATGAT-3′	qPCR (amplicon: 140 bp)	
S2^a^	(FAM)5′-TCTGATTGGTGCGCTGGTGGTCTGG-3′(TAMRA)		

trfA2-1	5′-CGAAATTCRTRTGGGAGAAGTA-3′	Quantification of IncP-1 by	Götz et al. (1996)
trfA2-2	5′-CGYTTGCAATGCACCAGGTC-3′	qPCR (amplicon: 241 bp)	

338F	5′-CCTACGGGAGGCAGCAG-3′	Quantification of 16S rDNA	Muyzer et al. (1993)
518R	5′-ATTACCGCGGCTGCTGG-3′	by qPCR (amplicon: 181 bp)	
			
968F-GC^b^	5′-CGCCCGCCGCGCCCCGCGCCCGTCCCGCCGCCCCCG	PCR for TTGE community	Cébron et al. (2009)
	CCCGAACGCGAAGAACCTTAC-3′	analyses	
1401R	5′-CGG TGT GTA CAA GAC CC-3′		

*a: Taqman^®^ probes; b: the GC clamp is underlined.*

Plasmids belonging to the IncP-1α/β were quantified by qPCR, using the “Power SYBR^®^ Green PCR Master Mix” (Applied Biosystems) with the primer set trfaA2-1/trfA2-2 (**Table 1**). As before, reactions were carried out in triplicate, using a “Step One Plus Real-Time PCR System” (Applied Biosystems, driver: StepOne Software v2.2), in a 25 μL volume with 25 ng of community DNA, 1 μM of each primer, and with thermocycling conditions set as follows: 10 min at 95°C followed by 45 cycles of 15 s at 95°C and 1 min at 60°C. The quality of the PCR products was subsequently checked by melting curve analyses, for which the temperature was ramped between 60°C and 95°C in increments of 0.3°C. IncP-1α/β quantitative results were normalized to the amount of eubacterial 16S rDNA, also quantified by qPCR with the 338F/518R universal primers using the same cycling conditions. For both 16S rDNA and IncP-1α/β quantifications, plasmid pBELX linearized with *BamH*1 was used as standard. In addition to pB10 DH5α target DNAs, plasmid pBELX also contains a synthetic sequence (Eurofins, France) covering part of the *E.coli* MG1655 16S rRNA gene (coordinates: 338–534; GenBank NR_102804.1) and part of the *trfA1* gene of plasmid pB10 (coordinates: 62887–63127; GenBank: AJ564903.1), here used as targets for qPCR.

Performance of qPCR assays in terms of specificity, efficiency, reproducibility and sensitivity (limit of detection) respected the instructions proposed by the MIQE guidelines (Minimum Information for Publication of Quantitative Real-Time PCR Experiments; Bustin et al., 2009). Typical standard curves obtained for qPCR targeting plasmid pB10, *E. coli* DH5α DNA, IncP-1α/β plasmids and eubacterial 16S rDNA genes are presented in supplementary material (Figure S1). Whatever the targeted DNA, qPCR assays were validated only if the amplifications from all the no-template controls were negative.

### TEMPORAL TEMPERATURE GRADIENT GEL ELECTROPHORESIS (TTGE)

Bacterial 16S rRNA gene fragments were amplified by PCR from community DNA extracts using the universal primer set 968F-GC and 1401R (**Table 1**). PCR amplification (using Taq DNA polymerase from Fermentas) and TTGE (performed on DCode system; Bio-Rad, France) were carried out according to Cébron et al. (2011). DNA banding patterns were visualized by SYBR Gold staining and analyzed on a GelDoc transilluminator (Bio-Rad). Image treatment and analysis were carried out using the QuantyOne 4.0.1 (Bio-Rad) coupled software, allowing cluster analysis using the unweighted pair group method with arithmetic averages (UPGMA) based on Jaccard similarity coefficients, which provide a percentage similarity between the fingerprints by pair wise comparison of TTGE band presence and absence. Community similarities were determined by Principal Component Analysis (PCA) of the relative band intensity of the TTGE profiles, with ADE-4 software (Thioulouse et al., 1997).

### STATISTICAL ANALYSES

The ability of each environmental matrix to support the dissemination of plasmid pB10 was deduced from the comparison of pB10 and *E. coli* DH5α DNAs relative stabilities in microcosms overtime (rate of disappearance). Transfer of pB10 was considered as effective when pB10 stability appeared significantly greater than the stability of its initial donor host (by at least a factor two). Microcosms were sorted in two groups according to whether transfer took place or not (without considering any rate of transfer), and both groups were then compared to each other with respect to their IncP-1 plasmid content. Considering (i) that the relative abundance of IncP-1α/β plasmids are quantitative values fitting a lognormal distribution, (ii) that each microcosms were performed independently, (iii) that the two groups of environmental matrices with respect with pB10 transfer are small size samples (De Winter, 2013) and, (iv) that the variances of these two groups were confirmed equal by using the Bartlett’s test, the Student’s *t*-test was used to determine if the data from these two groups are significantly different from each other. Statistical analyses were performed using the web tool BiostaTGV (http://marne.u707.jussieu.fr/biostatgv/).

## RESULTS

### MICROBIAL COMMUNITY STRUCTURE AND TRANSFER OF pB10

**FIGURE 1:**
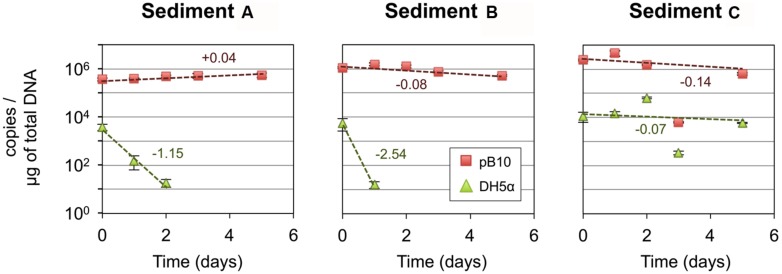
**Stability of plasmid pB10 and *Escherichia coli* DH5α in three river sediment microcosms (A–C)**. Numbers indicate rate of disappearance (expressed in log per day) calculated from an exponential curve fit of the data (dotted lines). Each data point is an average of three values obtained from triplicate qPCR reactions, error bars represent standard deviation. Part of the data were presented previously in Bonot and Merlin (2010).

**FIGURE 2:**
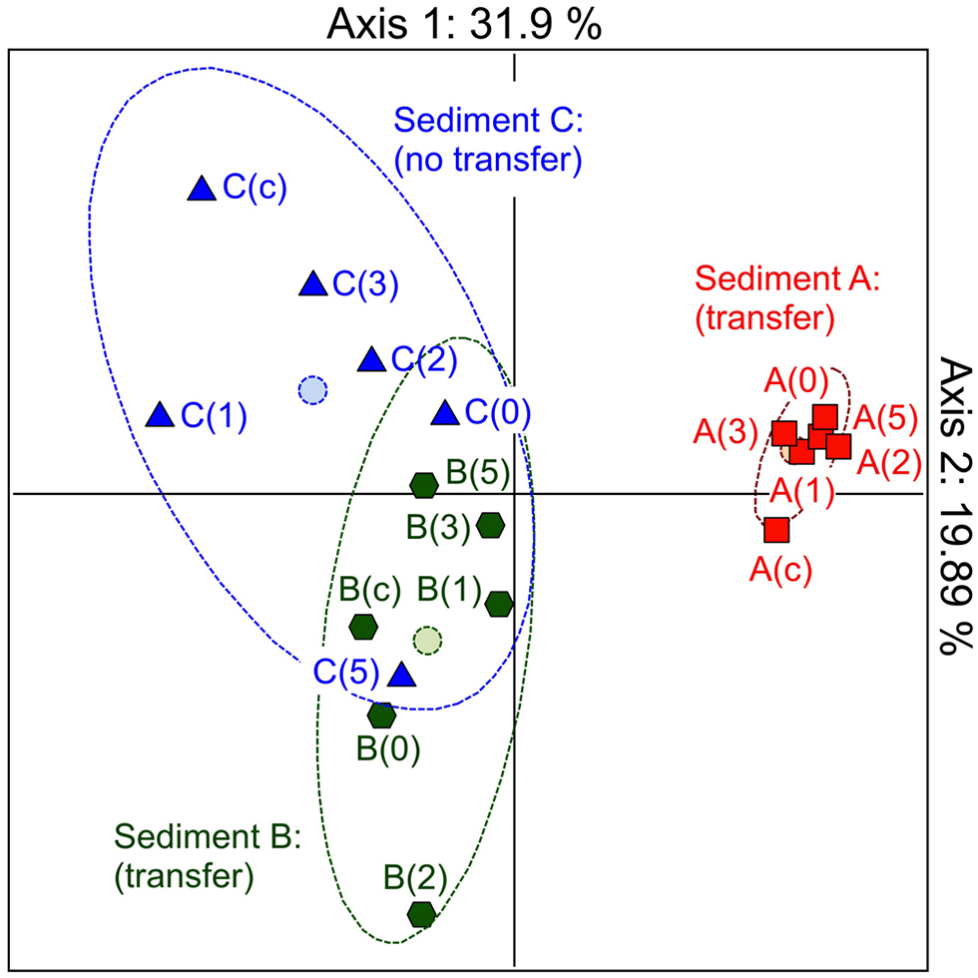
**Monitoring the community structures of sediment microcosms by TTGE of PCR amplified 16S rRNA genes.** Principal component analysis of band intensity data from TTGE profiles. Sediment microcosms (A–C) where sampled before or 0, 1, 2, 3, and 5 days after inoculation with *E. coli* DH5α(pB10).

In the first instance, we hypothesized that depending on its structure, the microbial community involved as recipient could be responsible for the efficiency of the pB10 dissemination in a given matrix. In order to highlight the involvement of particular community members, we compared the bacterial community structures of different but related microcosm samples, as well as their ability to support pB10 transfer. Three microcosms were set up with river sediments sampled simultaneously c.a. 50 m apart from each other but possibly impacted by different local inputs, which hopefully could provide some variability in terms of community structures and plasmid transfer. Sediment A, B, and C were sampled downstream a sewer overflow, upstream and downstream a WWTP discharge, respectively. Transfer experiments were initiated by inoculating the donor bacteria *E. coli* DH5α(pB10), and the relative abundances of both pB10 and *E. coli* DH5α DNA were monitored over time by qPCR. As previously described, for two sediment microcosms (A and B on **Figure 1**) pB10 appeared relatively stable while the donor host DH5α disappeared within 2 days, thus indicating that plasmid dissemination had occurred (Bonot and Merlin, 2010). Conversely, for the third sediment microcosm (sediment C), both DH5α and pB10 appeared more stable overtime and no transfer could be detected (**Figure 1**). The microcosm community structures and their evolution over time were studied by TTGE fingerprint analyses. A PCA was performed using the band intensity data of the TTGE profiles. The two principal components accounted for about 52% of the total variance. The ordination plot generated clearly shows a separation of the samples on the basis of their microcosm origins (**Figure 2**). The principal component one consistently separates the data point’s representative of the sediment A communities from the other two. To a lesser extent, the principal component two better discriminates communities from sediment B and C microcosms, which presented overlapping data points. Clearly, the ability of the sediment communities to support pB10 transfer (sediments A and B) does not seem to match the apparent community structure relatedness (sediments B and C), signifying that the TTGE is not resolvent enough to highlight a particular community member or a community structure that supposedly support the transfer of pB10.

### TRANSFER OF PLASMID pB10 AND OCCURRENCE OF IncP-1α/β PLASMIDS IN THE ENVIRONMENTAL COMMUNITIES

**FIGURE 3:**
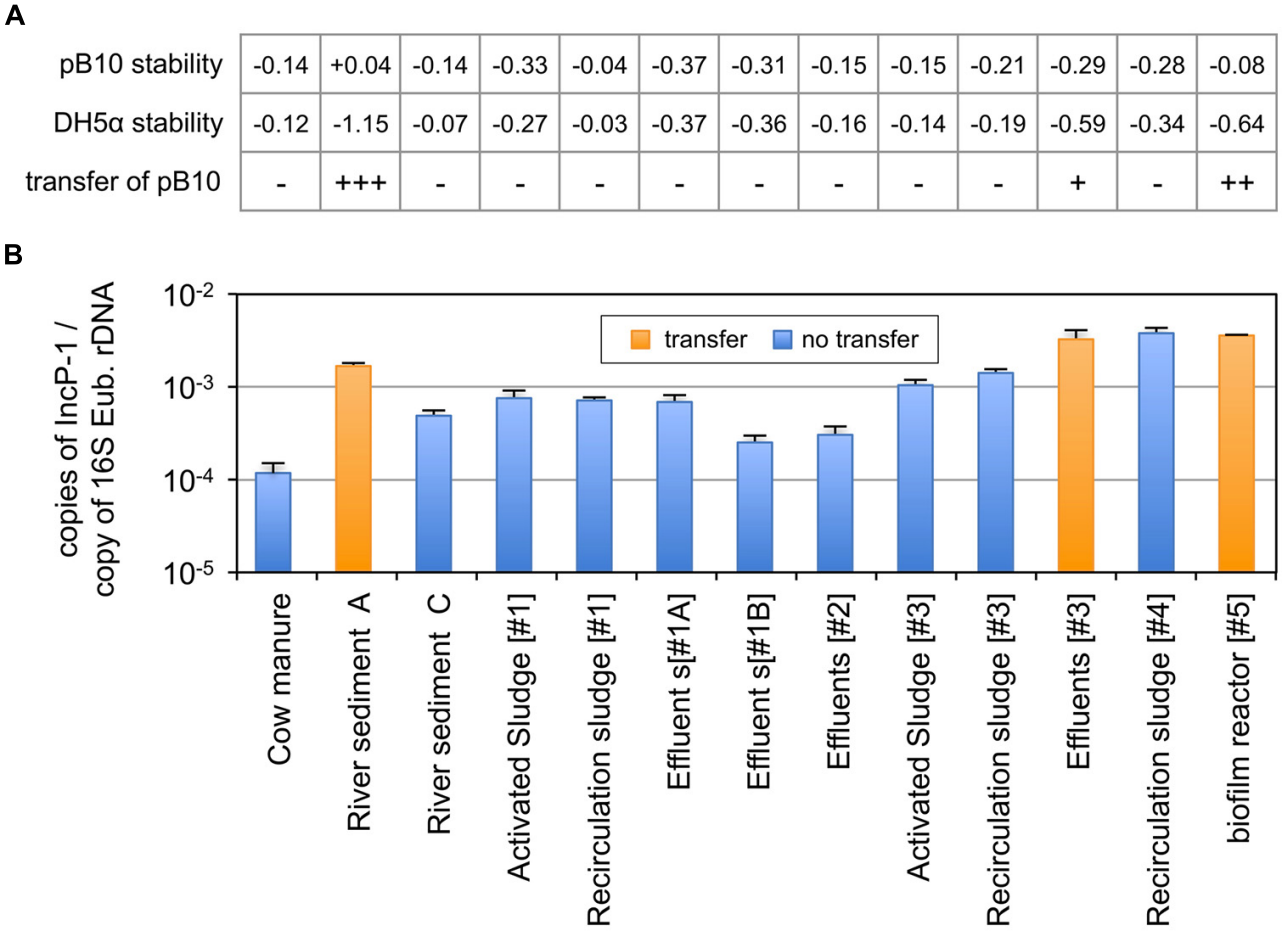
**Dissemination of plasmid pB10 (A) and relative abundance of IncP-1α/β plasmids (B) in various environmental communities maintained in microcosms.** The ability of each environmental matrix to support the dissemination of plasmid pB10 was deduced from the comparison of pB10 and *E. coli* DH5α DNA relative stabilities in microcosms overtime (rate of disappearance), as exemplified in **Figure 1** for sediments A–C. Transfer of pB10 was considered as effective (+) when pB10 stability appeared significantly greater than the stability of its initial donor host. Close rate of disappearance for both pB10 and *E. coli* DH5α reflects an absence of transfer (-). Values between brackets represent the number of the corresponding WWTPs. effluents [#1] were sampled twice in May 2011(#1A) and February 2012 (#1B). Each data point is an average of 3 values obtained from triplicate qPCR, where error bars represent standard deviation.

The combination of plasmid incompatibility and exclusion processes has long been recognized to reduce significantly the efficiency of conjugative transfer (Garcillán-Barcia and de la Cruz, 2008). We initially postulated that this so called “superinfection immunity” might play an important role in limiting the dissemination of plasmid pB10 in bacterial populations that were already hosting closely related IncP-1 plasmids. This hypothesis was tested by comparing the 13 environmental matrices for both their ability to support the transfer of plasmid pB10 and their content in IncP-1α/β plasmids. Microcosms were set up with environmental samples collected from (i) farm (manure), (ii) river (sediments), (iii) three different slaughterhouse WWTPs (activated sludge, recirculation sludge, effluents), and (iv) two different municipal WWTPs (recirculation sludge and biofilms from moving bed reactor). The microcosms were inoculated with *E. coli* DH5α(pB10), and the amount of both pB10 and *E. coli* DH5α DNA were monitored by qPCR over time. The disappearance rates of both DNAs were compared, from which the transfer of pB10 could be deduced (**Figure 3A**). Out of the 13 microcosms run, only three communities appeared to support the transfer of pB10, namely: the river sediment A, the biofilm from a WWTP moving bed reactor, and to a lesser extent the eﬄuent of one of the WWTPs. On the other hand, community DNAs extracted from microcosm samples collected before inoculation with the donor bacteria were used to assess the relative amount of plasmids belonging to the IncP-1α/β group. Against all odds, the three communities that where shown to support the transfer of pB10 also displayed the highest levels of IncP-1α/β plasmids (∼1.10^-3^–5.10^-3^ copies per 16S rDNA), while the non-permissive communities could present up to ten times less plasmids of that incompatibility group (**Figure 3B**). Based on the relative abundance of IncP-1α/β, the environmental matrices supporting the transfer of pB10 transfer and those that did not support this dissemination constitute two statistically distinct groups (*p* ≤ 0.02; Student’s test). It appears thus that contrary to what was initially thought, a relatively high abundance of IncP-1α/β plasmids might well represent a microbial community trait evidencing its permissiveness regarding the dissemination of plasmids of the same incompatibility group.

## DISCUSSION

After decades of investigation, conjugative plasmids clearly appear as key mobile genetic elements promoting the evolution of bacterial genomes thanks to the acquisition of exogenous sequences by horizontal transfer. Yet, despite being obvious when analyzing acquired DNA sequences from bacterial isolates, gene transfer remains difficult to demonstrate in complex environments when it comes to work with natural bacterial communities. In this respect, the reduced plasmid transfer efficiency observed in environmental communities, compared to pure culture, highlights the importance of the environmental matrix to support (or not) plasmid dissemination (Top et al., 1990; de la Cruz-Perera et al., 2013). We believe that transfer efficiencies are even slightly biased by the high loads of plasmid donor inoculum that are commonly used when working with natural communities (Bellanger et al., 2014). This brought us to develop a qPCR-based method that appears sensitive enough to detect rare events of pB10 transfer in realistic conditions, where the indigenous microbial communities remained numerically dominant compared to the donor inoculum size. Using this approach we could demonstrate the transfer of plasmid pB10 in a relatively limited number of environmental matrices, such as river sediments, biofilm reactors, or WWTP effluents and even manure under particular conditions where eukaryotic predation has been inhibited (Bonot and Merlin, 2010; Merlin et al., 2011; Bellanger et al., 2014). It should be noticed that if it provides the advantage of analyzing plasmid transfer at the level of community DNA, and therefore overcomes the limitation associated to the culture of environmental microbes, this methodology remains constrained by the efficiency of the DNA extraction method that may be narrowed to a particular subset of the community. With this respect it might be of interest to extend the exhaustivity of the analyses using different community DNA extraction methods. Still, such an approach is already relatively time consuming and therefore not really adequate to screen environmental matrices for their propensity to support pB10 transfer on a large scale.

The surprisingly poor transferability of plasmid pB10 in microcosm experiments was further explored by studying two matrix-associated parameters which may influence the transfer of pB10, namely the microbial community structure and its content in IncP-1 plasmids. The TTGE approach we used could not provide a clear picture to explain why some matrices, more than others, could support the transfer of the plasmid. Indeed, it appeared that even if some sediments supported a relatively high level of pB10 transfer, it could not be attributed to the presence of specific ribotypes/members in the microbial community. Clearly, this means that the transfer of plasmid pB10 was probably linked to poorly represented community members that will remain difficult to identify.

We also tried to correlate the absence of pB10 transfer in some microcosm communities to the presence of IncP-1 plasmids that could be involved in a so-called “superinfection immunity” phenomenon. This phenomenon has been well documented for decades and explained well how exclusion and incompatibility could prevent the stable acquisition of plasmids in bacteria bearing a related element (Garcillán-Barcia and de la Cruz, 2008). To the best of our knowledge, such a phenomenon has never been studied at the community level where, at best, only predicting models have been discussed (van der Hoeven, 1984, 1986). Surprisingly, in our hands the analysis carried out showed that the highest IncP-1α/β contents could be observed for the communities supporting the transfer of pB10. This observation does not completely rule out the relevance of “superinfection immunity” in affecting the transfer of plasmids in complex communities but, (i) in their complexity, the microbial communities may also contain permissive indigenous recipients that do not host incompatible plasmids, and (ii) it is established that superinfection immunity only reduces the efficiency of transfer rather than preventing it. Thus, a high IncP-1 content in the indigenous communities might reflect a high level of possible hosts for pB10 rather than an important limitation to transfer. Considering that, even with a broad host range, plasmid pB10 may have preferential hosts (Suzuki et al., 2010), the relative abundance of IncP-1 plasmids may, by itself, reveal a particular community structure. Additionally, it is tempting to speculate that the presence of other IncP-1 plasmids in the recipient community could even favor pB10 plasmid transfer in terms of fitness cost. As a matter of fact, the acquisition of a plasmid necessarily implies additional physiological burden for the new host because of the maintenance/replication of plasmid DNAs, the production of plasmid proteins, and the expression of the plasmid accessory functions. This cost reduces the fitness of the new host in non-selective environments, which is unfavorable to maintaining the plasmid in the community unless compensatory mutations had occurred (Lenski, 1998; Maisnier-Patin and Andersson, 2004; Harrison and Brockhurst, 2012). Somehow, communities displaying elevated abundances of IncP-1 plasmids may, at the same time, be composed of members that already have compensatory mutations that may be fitter for supporting the acquisition of related plasmids. In any case, and to the best of our knowledge, direct evidence linking the permissiveness of a community to plasmid transfer and its content in plasmids belonging to the same family is provided for the first time. This confirms plasmid occurrence analyses as valuable steps for screening and identifying environmental matrices that support plasmid dissemination, and thus pinpointing environmental hotspots posing risk of antibiotic resistance gene dissemination.

## CONCLUSION

This study brings to light the relationship between the occurrence of IncP-1 plasmids in microbial communities and their permissiveness to plasmid transfer. Taken at the level of individuals, superinfection immunity limits the acquisition of plasmids related to resident elements, and therefore should limit plasmid dissemination. Nevertheless, our data demonstrate that, at community level, an elevated abundance of IncP-1 plasmids is far from being an obstacle to the dissemination of a plasmid belonging to the same group. To the contrary, it may even reflect a certain permissiveness of the community for the acquisition of plasmids belonging to that group. Back in the context of horizontal gene transfer, the relative abundance of plasmids appears as a community trait that could be useful to identify hot spots of antibiotic resistance gene spread in environmental settings. Considering how tedious the demonstration of plasmid transfer is in complex natural communities (Bellanger et al., 2014), evaluating plasmid abundance first could become an easy way to pre-screen environmental samples prior to demonstrating the effectiveness of plasmid dissemination in a selection of communities. Then, a wide scale environmental screening should lead to the identification of environmental stressors and selectors driving plasmid transfer, and ultimately allow taking action to better control plasmid-based dissemination of antibiotic resistance genes.

## Conflict of Interest Statement

The authors declare that the research was conducted in the absence of any commercial or financial relationships that could be construed as a potential conflict of interest.

## References

[B1] Bellanger X. Guilloteau H. Bonot S. Merlin C. (2014). Demonstrating plasmid-based horizontal gene transfer in complex environmental matrices: a practical approach for a critical review. *Sci. Total Environ.* 493 872–882 10.1016/j.scitotenv.2014.06.070 25000583

[B2] Bonot S. Courtois S. Block J.-C. Merlin C. (2010). Improving the recovery of qPCR-grade DNA from sludge and sediment. *Appl. Microbiol. Biotechnol.* 87 2303–2311 10.1007/s00253-010-2686-0 20563719

[B3] Bonot S. Merlin C. (2010). Monitoring the dissemination of the broad-host-range plasmid pB10 in sediment microcosms by quantitative PCR. *Appl. Environ. Microbiol.* 76 378–382 10.1128/AEM.01125-09 19897757 PMC2798623

[B4] Bustin S. A. Benes V. Garson J. A. Hellemans J. Huggett J. Kubista M. (2009). The MIQE guidelines: minimum information for publication of quantitative real-time PCR experiments. *Clin. Chem.* 55 611–622 10.1373/clinchem.2008.112797 19246619

[B5] Cébron A. Beguiristain T. Faure P. Norini M. P. Masfaraud J. F. Leyval C. (2009). Influence of vegetation on the in situ bacterial community and polycyclic aromatic hydrocarbon (PAH) degraders in aged PAH-contaminated or thermal-desorption-treated soil. *Appl. Environ. Microbiol.* 75 6322–6330 10.1128/AEM.02862-2868 19633127 PMC2753067

[B6] Cébron A. Louvel B. Faure P. France-Lanord C. Chen Y. Murrell J. C. (2011). Root exudates modify bacterial diversity of phenanthrene degraders in PAH-polluted soil but not phenanthrene degradation rates. *Environ. Microbiol.* 13 722–736 10.1111/j.1462-2920.2010.02376.x 21087382

[B7] Davies J. (1995). Vicious circles: looking back on resistance plasmids. *Genetics* 139 1465–1468. 7789752 10.1093/genetics/139.4.1465 PMC1206476

[B8] Davies J. (2007). Microbes have the last word. A drastic re-evaluation of antimicrobial treatment is needed to overcome the threat of antibiotic-resistant bacteria. *EMBO Rep.* 8 616–621 10.1038/sj.embor.7401022 17603533 PMC1905906

[B9] Davies J. Davies D. (2010). Origins and evolution of antibiotic resistance. *Microbiol. Mol. Biol. Rev.* 74 417–433 10.1128/MMBR.00016-10 20805405 PMC2937522

[B10] de la Cruz-Perera C. I. Ren D. Blanchet M. Dendooven L. Marsch R. Sørensen S. J. (2013). The ability of soil bacteria to receive the conjugative IncP1 plasmid, pKJK10, is different in a mixed community compared to single strains. *FEMS Microbiol. Lett.* 338 95–100 10.1111/1574-6968.12036 23106414

[B11] De Winter J. C. F. (2013). Using the Student’s t-test with extremely small sample sizes. *Pract. Assess. Res. Eval.* 18 2.

[B12] Garcillán-Barcia M. P. de la Cruz F. (2008). Why is entry exclusion an essential feature of conjugative plasmids? *Plasmid* 60 1–18 10.1016/j.plasmid.2008.03.002 18440635

[B13] Götz A. Pukall R. Smit E. Tietze E. Prager R. Tschäpe H. (1996). Detection and characterization of broad-host-range plasmids in environmental bacteria by PCR. *Appl. Environ. Microbiol.* 62 2621–2628. 8779598 10.1128/aem.62.7.2621-2628.1996 PMC168041

[B14] Harrison E. Brockhurst M. A. (2012). Plasmid-mediated horizontal gene transfer is a coevolutionary process. *Trends Microbiol.* 20 262–267 10.1016/j.tim.2012.04.003 22564249

[B15] Heuer H. Abdo Z. Smalla K. (2008). Patchy distribution of flexible genetic elements in bacterial populations mediates robustness to environmental uncertainty. *FEMS Microbiol. Ecol.* 65 361–371 10.1111/j.1574-6941.2008.00539.x 18616581

[B16] Heuer H. Kopmann C. Binh C. T. Top E. M. Smalla K. (2009). Spreading antibiotic resistance through spread manure: characteristics of a novel plasmid type with low %G+C content. *Environ. Microbiol.* 11 937–949 10.1111/j.1462-2920.2008.01819 19055690

[B17] Knapp C. W. Dolfing J. Ehlert P. A. Graham D. W. (2010). Evidence of increasing antibiotic resistance gene abundances in archived soils since 1940. *Environ. Sci. Technol.* 44 580–587 10.1021/es901221x 20025282

[B18] Kristiansson E. Fick J. Janzon A. Grabic R. Rutgersson C. Weijdegård B. (2011). Pyrosequencing of antibiotic-contaminated river sediments reveals high levels of resistance and gene transfer elements. *PLoS ONE* 6:e17038 10.1371/journal.pone.0017038 PMC3040208 21359229

[B19] Lenski R. E. (1998). Bacterial evolution and the cost of antibiotic resistance. *Int. Microbiol.* 1 265–270. 10943373

[B20] Maisnier-Patin S. Andersson D. I. (2004). Adaptation to the deleterious effects of antimicrobial drug resistance mutations by compensatory evolution. *Res. Microbiol.* 155 360–369 10.1016/j.resmic.2004.01.019 15207868

[B21] Merlin C. Bonot S. Courtois S. Block J.-C. (2011). Persistence and dissemination of the multiple-antibiotic-resistance plasmid pB10 in the microbial communities of wastewater sludge microcosms. *Water Res.* 45 2897–2905 10.1016/j.watres.2011.03.002 21440282

[B22] Muyzer G. de Waal E. C. Uitterlinden A. G. (1993). Profiling of complex microbial populations by denaturing gradient gel electrophoresis analysis of polymerase chain reaction-amplified genes coding for 16S rRNA. *Appl. Environ. Microbiol.* 59 695–700. 7683183 10.1128/aem.59.3.695-700.1993 PMC202176

[B23] Port J. A. Cullen A. C. Wallace J. C. Smith M. N. Faustman E. M. (2014). Metagenomic frameworks for monitoring antibiotic resistance in aquatic environments. *Environ. Health Perspect.* 122 222–228 10.1289/ehp.1307009 24334622 PMC3948035

[B24] Porteous L. A. Seidler R. J. Watrud L. S. (1997). An improved method for purifying DNA from soil for polymerase chain reaction amplification and molecular ecology applications. *Mol. Ecol.* 6 787–791 10.1046/j.1365-294X.1997.00241.x

[B25] Rizzo L. Manaia C. Merlin C. Schwartz T. Dagot C. Ploy M. C. (2013). Urban wastewater treatment plants as hotspots for antibiotic resistant bacteria and genes spread into the environment: a review. *Sci. Total Environ.* 447 345–360 10.1016/j.scitotenv.2013.01.032 23396083

[B26] Schlüter A. Heuer H. Szczepanowski R. Forney L. J. Thomas C. M. Pühler A. (2003). The 64 508 bp IncP-1β antibiotic multiresistance plasmid pB10 isolated from a waste-water treatment plant provides evidence for recombination between members of different branches of the IncP-1β group. *Microbiology* 149 3139–3153 10.1099/mic.0.26570-26570 14600226

[B27] Stalder T. Barraud O. Casellas M. Dagot C. Ploy M. C. (2012). Integron involvement in environmental spread of antibiotic resistance. *Front. Microbiol.* 3:119 10.3389/fmicb.2012.00119 PMC3321497 22509175

[B28] Suzuki H. Yano H. Brown C. J. Top E. M. (2010). Predicting plasmid promiscuity based on genomic signature. *J. Bacteriol.* 192 6045–6055 10.1128/JB.00277-210 20851899 PMC2976448

[B29] Thioulouse J. Chessel D. Dol´edec S. Olivier J. M. (1997). ADE-4: a multivariate analysis and graphical display software. *Stat. Comput.* 7 75–83 10.1023/A:1018513530268

[B30] Top E. Mergeay M. Springael D. Verstraete W. (1990). Gene escape model: transfer of heavy metal resistance genes from *Escherichia coli* to *Alcaligenes eutrophus* on agar plates and in soil samples. *Appl. Environ. Microbiol.* 56 2471–2479. 2206101 10.1128/aem.56.8.2471-2479.1990 PMC184750

[B31] Toussaint A. Chandler M. (2012). “Prokaryote genome fluidity: toward a system approach of the mobilome,” in *Bacterial Molecular Networks: Methods and Protocols*,*Methods in Molecular Biology* eds van Helden J. Toussaint A. Thieffry D. (New York, NY: Humana Press) 804 57–80 10.1007/978-1-61779-361-5_4 22144148

[B32] van der Hoeven N. (1984). A mathematical model for the co-existence of incompatible, conjugative plasmids in individual bacteria of a bacterial population. *J. Theor. Biol.* 110 411–423 10.1016/S0022-5193(84)80183-6 6503308

[B33] van der Hoeven N. (1986). Coexistence of incompatible plasmids in a bacterial population living under a feast and famine regime. *J. Math. Biol.* 24 313–325 10.1007/BF00275640 3531375

[B34] Wang Z. Zhang X. X. Huang K. Miao Y. Shi P. Liu B. (2013). Metagenomic profiling of antibiotic resistance genes and mobile genetic elements in a tannery wastewater treatment plant. *PLoS ONE* 8:e76079 10.1371/journal.pone.0076079 PMC3787945 24098424

[B35] Wiedenbeck J. Cohan F. M. (2011). Origins of bacterial diversity through horizontal genetic transfer and adaptation to new ecological niches. *FEMS Microbiol. Rev.* 35 957–976 10.1111/j.1574-6976.2011.00292.x 21711367

[B36] Wright G. D. (2010). Antibiotic resistance in the environment: a link to the clinic? *Curr. Opin. Microbiol.* 13 589–594 10.1016/j.mib.2010.08.005 20850375

