# Supplementary material for: Natural microbial communities supporting the transfer of the IncP-1β plasmid pB10 exhibit a higher initial content of plasmids from the same incompatibility group
Source: Front Microbiol. 2014 Nov 24;5:637. doi: 10.3389/fmicb.2014.00637 (PMC4241820; doi:10.3389/fmicb.2014.00637)
Supplement: Supplementary file 1 [file Data_Sheet_1.DOCX]

Supplemental material

**Natural microbial communities supporting the transfer of the IncP-1β**

**plasmid pB10 exhibit a higher initial content of plasmids**

**from the same incompatibility group**

**Xavier Bellanger, Hélène Guilloteau, Bérengère Breuil, Christophe Merlin**

1. **Characteristics of the environmental matrices used in this study**

## Sampling sites

Environmental samples were collected from different sampling sites, some of which were described previously. All samples were used fresh for conducting pB10 transfer experiments in microcosms, while quantification of IncP-1 plasmids was carried out on total community DNA stored frozen.

Samples Sampling sites References

Cow manure Prim’Holstein dairy farm close to Nancy Bellanger et al., 2014

(Lorraine, France).

River sediments River sediments A, B and C were collected 50 m Bonot et al., 2010

apart from each other in river Moselle at Golbey

(Lorraine, France).

WWTP samples [#1] Slaughterhouse WWTP [#1], dedicated to beef, This study

sampled for activated sludge, recirculation sludge,

and effluents (North-West of France).

WWTP samples [#2] Slaughterhouse WWTP [#2], dedicated to beef, This study

sampled for effluents (North-West of France).

WWTP samples [#3] Slaughterhouse WWTP [#3], dedicated to calves, Bellanger et al., 2014

sampled for activated sludge, recirculation sludge,

and effluents (North-West of France).

WWTP samples [#4] Municipal WWTP [#4] from the area of Nancy This study

(France), sampled for recirculation sludge.

WWTP samples [#5] Municipal WWTP [#5], colonized polyethylene disks Merlin et al., 2011

sampled in an aeration tank (moving bed reactor).

*WWTP: Wastewater Treatment Plant*

## Cow manure characteristics

Fresh samples were obtained in May 2013 from a collector receiving fresh manure continuously scraped from a diary cow barn. Manure characteristics obtained from a unique sample were as follows: solid material: 11.6% (mass); pH: 7.45; conductivity: 10.97 mS.cm^-1^; dissolved O_2_: 0.28 mg.L^-1^ (Bellanger et al., 2014).

## River sediment characteristics

River sediments were collected in March 2009 in the river Moselle at Golbey (Lorraine, France). A global sediment analysis, carried out by the LIEC (University of Lorraine, France), was reported previously (Bonot et al., 2010). It showed the following chemical composition: 78% wt SiO2, 9% wt Al_2_O_3_, 3% wt Fe_2_O_3_, 3.5% wt K_2_O, 1% wt MgO, and finally 2% wt loss on ignition which can be attributed to natural organic matter; it also displayed the following particle size distribution: 30% wt greater than 2 mm, 64.5% wt in the 250 μm–2 mm fraction, 5% wt in the 50–250 μm fraction, 0.5% wt less than 50 μm. Sediments A, B, and C were collected 50 meters apart from each other, downstream a sewer overflow (A), upstream a WWTP (B) and downstream a WWTP discharge (C), respectively.

## Slaughterhouse WWTP sample characteristics

Three slaughterhouse WWTPs form the North-West of France were sampled independently for either activated sludge, recirculation sludge or effluents. Activated sludge were sampled in biological basins, recirculation sludge were sampled just before re-introduction in biological basins, and effluents were recovered from automatic samplers operating for 24 h. Collected samples presented the following characteristics:

**WWTP [#1] WWTP [#2] WWTP [#3]**

Samples Activated Recycle Effluents Effluents Activated Recycle Effluents

sludge sludge sludge sludge

pH 7.3 7.2 7.6 nd 7.0 7.2 7.1

TSS (g.L^-1^) 8.1 10.7 1.4 nd 9.9 9.3 3.8

VSS (g.L^-1^) 5.5 7.6 0.0 nd 4.3 4.5 0.1

Dissolved O_2_ 0.1 0.1 7.0 nd 0.1 0.1 3.8

(mg.L^-1^)

*TSS: total suspended solids; VSS: volatile suspended solids; nd: not done*

WWTP [#1] was sampled in May 2011 and an additional effluent sample was collect in February 2012 (effluent sample [#1]B). WWTP [#2] and [#3] were sampled in April 2011 and February 2012, respectively.

## Municipal WWTP sample characteristics

WWTP [#4] (500,000 population equivalent) was sampled in May 2012 to collect recirculation sludge. Additional information regarding WWTP [#4] can be obtained in Pasquini et al. (2013) and Pasquini et al. (2014).

Polyethylene disks colonized by biofilms were collected in the moving bed reactor (aeration tank) of WWTP [#5] (250 population equivalent) in April 2009. Biofilm samples were described previously (Merlin et al., 2011).

1. **Compliance to MIQE Guidelines for qPCR (Bustin et al., 2009)**

*Compliance to MIQE Guidelines for qPCR (continued)*

**Figure S1. Standard curves corresponding to the different qPCR assays used in the study.** The source of the DNA used as standard for the measurements are indicated between brackets. The value reported for each quantification cycle is the mean of 3 to 4 independent experiments and therefore present intra-assay variations (error bars represent standard deviations). It should be noticed that, although DH5α and pB10 DNA were quantified using standard DNA from different sources, the standard curves were similar in pairs.

**Fig. S2.** Melting curves for qPCR amplification products obtained during the quantification of IncP-1α/1β plasmids using the primer set trfaA2-1/trfA2-2 (SYBR green Technology). **A:** melting curves of qPCR products obtained using pB10 as template; **B:** melting curve of the qPCR product obtained using total community DNA as template.

1. **References**

Bellanger, X., Guilloteau, H., Bonot, S., and Merlin C. (2014) Demonstrating plasmid-based horizontal gene transfer in complex environmental matrices: a practical approach for a critical review. *Sci. Total Environ.* 493C,872-882. (doi: 10.1016/j.scitotenv.2014.06.070)

Bonot, S., and Merlin, C. (2010). Monitoring the dissemination of the broad-host-range plasmid pB10 in sediment microcosms by quantitative PCR. *Appl. Environ. Microbiol.* 76,378-382. (doi: 10.1128/AEM.01125-09)

Bonot, S., Courtois, S., Block, J.-C., and Merlin, C. (2010). Improving the recovery of qPCR-grade DNA from sludge and sediment. *Appl. Microbiol. Biotechnol.* 87, 2303-2311. (doi: 10.1007/s00253-010-2686-0)

Bustin, S. A., Benes, V., Garson, J. A., Hellemans, J., Huggett, J., Kubista, M., Mueller R., Nolan T., Pfaffl M. W., Shipley G. L., Vandesompele J., and Wittwer, C. T. (2009). The MIQE guidelines: minimum information for publication of quantitative real-time PCR experiments. *Clinical chemistry*, *55*(4), 611-622. (doi: 10.1373/clinchem.2008.112797)

Merlin, C., Bonot, S., Courtois, S., and Block, J.-C. (2011). Persistence and dissemination of the multiple-antibiotic-resistance plasmid pB10 in the microbial communities of wastewater sludge microcosms. *Water Res.* 45,2897-2905. (doi: 10.1016/j.watres.2011.03.002)

Pasquini, L., Merlin, C., Hassenboehler, L., Munoz, J.-F., Pons, M.-N., and Görner, T. (2013). Impact of certain household micropollutants on bacterial behavior. Toxicity tests/study of extracellular polymeric substances in sludge. *Sci. Total Environ.* 463-464,355-65. (doi: 10.1016/j.scitotenv.2013.06.018)

Pasquini, L., Munoz, J.-F., Pons, M.-N., Yvon, J., Dauchy, X., France, X., Le N.D., France-Lanord, C., Görner, T. (2014). Occurrence of eight household micropollutants in urban wastewater and their fate in a wastewater treatment plant. Statistical evaluation. *Sci. Total Environ.* 481,459-68. (doi: 10.1016/j.scitotenv.2014.02.075)
